# Factors Affecting Voluntary Breath-Holding Duration and Breaking Point in Young Adults

**DOI:** 10.4314/ejhs.v34i3.7

**Published:** 2024-05

**Authors:** Rahaf MohammedRashad Marwah, Norah Asem Alsulami, Raghad Saleh Alyami, Mohamed Eldigire Ahmed, Raju Suresh Kumar

**Affiliations:** 1 Department of Respiratory Therapy, College of Applied Medical Sciences; 2 Department of Basic Sciences, College of Science and Health Professions; 3 King Saud bin Abdulaziz University for Health Sciences, Jeddah, Saudi Arabia; 4 King Abdullah International Medical Research Center (KAIMRC), Jeddah, Saudi Arabia

**Keywords:** Breath-holding duration, breaking point, isometric handgrip test, cold pressor test, mental arithmetic task

## Abstract

**Background:**

Studies have reported that breath-holding duration (BHD) in humans varies and can be influenced by several factors. The breaking point is an involuntary respiratory act that overrides the urge to hold one's breath, preventing unconsciousness. This study investigated the effects of the isometric handgrip test, cold pressor test and mental arithmetic task on BHD and its breakpoint in young, healthy Saudi Arabian subjects.

**Methods:**

This interventional study, with a single-arm design, involved 78 young adults. Before conducting breath-holding duration (BHD) testing, their age, gender, height, weight, and systolic and diastolic blood pressure were recorded. BHD was assessed under various experimental conditions, such as rest, during an isometric handgrip test, a cold pressor test, and mental arithmetic tasks. Blood pressure (BP) and oxygen saturation (SpO2) levels were continuously monitored.

**Results:**

The study subjects had a mean age of 21.23 years, with 47 (60.3%) females and 31 (39.7%) males. The mean Body Mass Index was 24.14 kg/m2. During rest, the mean BHD value was 29.67 seconds, while it was 26.21 seconds during isometric handgrip. The cold pressor test recorded a mean value of 32.69 seconds, and the mental arithmetic test recorded a mean value of 33.08 seconds.

**Conclusion:**

The findings of this study indicate a reduction in BHD during isometric handgrip and augmentation during the cold pressor and mental arithmetic tests.

## Introduction

Breath-holding is a voluntary act that can increase carbon dioxide levels in the blood and lungs to a point known as the “breaking point”. At this point, the body's instinct to breathe takes over, ensuring that the body gets enough oxygen to prevent unconsciousness. This involuntary respiratory act overrides voluntary breath-holding and helps to maintain the body's oxygen levels (1, 2).

Holding the breath reduces PaO2 levels while tissue oxygen consumption continues (3). The average breath-holding duration (BHD), according to researchers, appeared to be a mean value of 78 seconds when holding the breath at total lung capacity (TLC) with a variable range of (72-78) seconds(4). Researchers have discovered that breath-holding duration can vary significantly based on several factors, including individual differences and fluctuations within the same person. A research study reported a 138-sec increase in the BHD due to hyperventilation, thereby lowering the arterial PCO_2_ and a 157s increase in breath-holding duration by increasing the arterial PO_2_ (5). According to M.J. Parkes, mental distraction can increase breath-holding duration by 13-19%. Moreover, successive trials in the same individual can increase the voluntary breath-holding duration by up to 37% (1).

Studies have suggested a potential link between psychological factors and BHD (3,6). Investigators have revealed a close association between mental status and its interaction with the locus coeruleus (LC), a pontine nucleus that regulates respiration. Ultimately, changes in mental status can cause changes in respiration (7). One of the aims of our study was to explore how mental arithmetic tasks in the participants would affect their BHD. Furthermore, changes in hemodynamic status can impact respiration and vice versa. Notably, hemodynamic alterations can lead to respiratory changes and affect hemodynamics (8).

The Cold Pressor Test (CPT) is a globally accepted method for researchers in evaluating autonomic functions (9). It has been reported that CPT can significantly impact blood pressure. It acts primarily through stimulation of the sympathetic nervous system (10). The isometric hand grip strength test is a cardiopulmonary function predictor (11).

One of the aims of our study was to understand whether an isometric handgrip test has any effect on the BHD. Although there are few studies available in the literature that explain the role of multiple factors in voluntary breath-holding duration and its breaking point (1,5), no correlative research has been conducted to investigate the influence of the isometric handgrip test, CPT, and mental arithmetic test on the BHD. To the best of our knowledge, no such correlative studies on BHD have been carried out on healthy young adults. The lacuna in existing literature emphasizes the necessity for additional research in this field. Therefore, our study aimed to investigate the effects of isometric handgrip tests, mental arithmetic tasks, and cold pressor testson BHD in healthy young adult Saudi Arabian subjects.

## Methods

**Study design and setting**: This single-arm interventional study was conducted in the Respiratory Therapy Department of the College of Applied Medical Sciences (CAMS) at King Saud bin Abdulaziz University for Health Sciences (KSAU-HS) in Jeddah, Saudi Arabia.

**Study population and eligibility**: A total of 343 male and female students, aged between 19 and 23 years, who were enrolled in various health sciences programs at the university, were screened to obtain the required sample size. The study was conducted over a period of one year. The inclusion criteria for the study were healthy students with no history of illnesses. Subjects with hypertension, injuries or weakness in the upper limbs, motor neuron diseases, diabetes mellitus, respiratory diseases, smokers, fasting, and pregnant subjects were excluded from the study.

**Sample size determination**: As mentioned in another study (12), the sample size was calculated using the sample size formula for estimating the population mean. Using a 95% confidence level, a 2% margin of error, and an 8.7 standard deviation of BMI, the required sample size was calculated to be 72 (n=72).

**Sampling technique**: Consecutive sampling, a non-probability sampling technique, was employed in this study, where subjects were conveniently selected until the desired sample size was achieved. The demographic information included height and weight, age and gender, and BMI. Also, blood pressure (BP) and oxygen saturation (SpO_2_) were taken as independent variables, while the dependent variable was BHD in seconds. The data was collected using a form that included the study variables. The subject's height and weight were monitored and recorded before the start of the tests. The BHD was measured during the isometric handgrip test, cold pressor test, mental arithmetic test, and at rest. Throughout the tests, the subjects' BP and SpO_2_ were monitored. The isometric handgrip test was performed using an isometric handgrip (Generic Hand Grip, Saudi Arabia). A sphygmomanometer (KBM, model DSK-1011, Japan), a pulse oximeter (iCare modelAt101C, Taiwan), and a stopwatch were used in the experiments.

**Ethical approval**: Approval was obtained from the Institutional Review Board (Refno: SP 20/355/J) of King Abdullah International Medical Research Center (KAIMRC) for the research proposal, methodology, data collection procedures, and consent forms used in this study. The study complied with ethical standards set by the institutional research committee, including those outlined in the 1964 Helsinki Declaration and its subsequent amendments. At the beginning of the experiment, written approval in the form of signed consent was obtained from all the participants included in the study. During the investigation, we ensured to protect the subject's privacy. Any personal information related to the subject was kept confidential. At the start of the experiment, we recorded the subjects' resting BHD, blood pressure, blood oxygen saturation, and respiratory rate.

**Isometric hand grip test**: This test was performed using the dominant hand for the subject. The blood pressure cuff was tied appropriately over the subject's upper arm of the non-dominant hand, positioning on the brachial artery. SpO2 levels were measured from the non-dominant hand during the experiment. Subjects were instructed to hold an isometric grip in their dominant hand with their arm supported on a table. The subject's wrist was kept in a neutral position while the elbow was bent at a 90° angle. They were asked to perform one maximum voluntary contraction using the isometric handgrip and keep it pressed for at least one minute (13). During this time, the subjects were asked to perform voluntary breath-holding after maximum inspiration and hold their breaths for the maximum possible duration until they reached the breaking point, where they could no longer hold their breath. The BHD was measured in seconds using a stopwatch. The starting time was noted from the peak of inhalation, while the end time was determined by the moment of breath release. Systolic and diastolic blood pressure was monitored during the procedure.

**Cold pressor test (CPT)**: Participants in the study were instructed to immerse the palm of their dominant hand into an ice-cold water bath, with a temperature ranging from 3 to 5 °C, just above the wrist for one to two minutes (14). After being immersed in the ice-cold water bath, the participants were instructed to hold their breath as long as possible until they reached their breaking point. They were allowed to remove their hands from the water if they felt uncomfortable or unable to withstand the cold sensation. BP and SpO2 were simultaneously measured from their non-dominant hand with the cold pressor test. BHD was noted in seconds.

**Mental arithmetic test**: Studies have shown that mental arithmetic stress can lead to changes in cardio-respiratory responses, resulting in alterations in blood pressure and BHD (5, 15). During our research, we asked the subjects to hold their breath for as long as possible while solving four mental arithmetic tasks. The participants were given a set of mental arithmetic tasks, which included addition, subtraction, multiplication, and division of two-digit numbers. They were shown a chart board with the correct answers and asked to choose the right answer from three options using their dominant hand's index finger. All participants were given the same problems to maintain consistency and the same difficulty level. While the subjects were solving the problems, we measured their non-dominant hand's BP and SpO2. BHD was recorded in seconds.

**Data Analysis**: The data was carefully cleaned and checked for accuracy before being entered into statistical software. Excel and SPSS 25.0 software (V.25; IBM, Chicago, Illinois, USA) were used for data analysis. Descriptive statistics such as means, standard deviation, variations, frequency, and percentage distribution were calculated for the study variables. Correlation and regression tests were conducted to examine the differences between the two datasets. A p-value of less than or equal to 0.05 was considered significant.

## Results

Seventy-eight healthy participants (mean age: 21.23 ± 0.73) were included. Of the subjects. 47(60.3%) were females, and 31(39.7%) were males. The average BMI was 24.14 kg/m2, with a standard deviation 5.24. The means and standard deviations of all parameters measured in the study are represented in Table 1. BHD was compared to isometric handgrip, CPT, mental arithmetic tests and during resting conditions.

**Table 1:** Descriptive statistics of the different parameters measured in the study

Parameters		N	Mean	Standard Deviation
**Breath-holding** **duration (BHD) in seconds**	At rest			
	During isometric handgrip	78	26.21	11.55
	During the cold pressor test	78	32.69	12.78
	During the mental arithmetic task	78	33.08	12.94
	Total	312	30.41	12.85
**Systolic BP** **mm Hg**	At rest	78	122.28	13.43
	During isometric handgrip	78	134.67	18.05
	During the cold pressor test	78	132.35	17.23
	During the mental arithmetic task	78	127.75	17.19
	Total	312	129.26	17.16
**Diastolic BP** **mm Hg**	At rest	78	74.58	9.42
	During isometric handgrip	78	89.26	15.94
	During the cold pressor test	78	83.57	13.63
	During the mental arithmetic task	78	82.06	14.13
	Total	312	82.37	14.42
**Oxygen saturation (SpO2)** **%**	At rest	78	98.35	0.010
	During isometric handgrip	78	98.28	0.012
	During the cold pressor test	78	98.10	0.012
	During the mental arithmetic task	78	98.42	0.011
	Total	312	98.29	0.011

**Figure 1:**
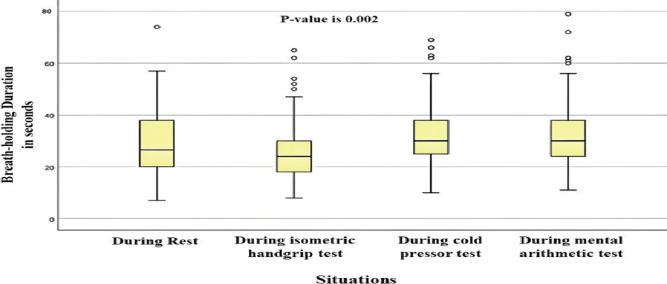
Comparison of BHD during various situations

Results show that BHD was highest during mental arithmetic tests, and BHD was lowest during the isometric handgrip test, as shown in (Figure 1). A correlation coefficient test (bivariate analysis) determined the correlation between BHD, isometric handgrip, CPT, and mental arithmetic tests. Table 2 shows that BHD significantly correlates with systolic and diastolic blood pressure during an isometric handgrip test. Moreover, breath-holding duration also significantly correlates with systolic blood pressure during mental arithmetic tasks.

**Table 2:** Correlation between breath-holding duration (BHD), Systolic BP, Diastolic BP & Oxygen saturation (SpO2)

Situations			Breath-holding duration in seconds
**At rest**	Systolic BP		0.173
		Sig. (2-tailed)	0.130
	Diastolic BP		-0.091
		Sig. (2-tailed)	0.426
	Oxygen saturation (SpO2)		0.144
		Sig. (2-tailed)	0.210
**During isometric handgrip**	Systolic BP		0.378**
		Sig. (2-tailed)	0.001
	Diastolic BP		0.230*
		Sig. (2-tailed)	0.043
	Oxygen saturation (SpO2)		0.055
		Sig. (2-tailed)	0.634
**During the cold pressor test**			
	Systolic BP		0.196
		Sig. (2-tailed)	0.085
	Diastolic BP		-0.040
		Sig. (2-tailed)	0.731
	Oxygen saturation (SpO2)		0.039
		Sig. (2-tailed)	0.734
**During mental arithmetic task**	Systolic BP		0.240*
		Sig. (2-tailed)	0.035
	Diastolic BP		0.186
		Sig. (2-tailed)	0.102
	Oxygen saturation (SpO2)		-0.139
		Sig. (2-tailed)	0.224

** Significant at %

* Significant at 5%

A regression analysis determined the relationship between BHD and systolic BP, diastolic BP, and SpO2 in the same subject. Table 3 shows that systolic blood pressure had a significant P-value in all tests except for mental arithmetic tasks. Results of the study showed a correlation between systolic blood pressure and three tests: the isometric handgrip test, CPT, and mental arithmetic test. The p-value was 0.006 at rest, 0.006 during the isometric handgrip test, and 0.018 during the cold pressor test. The systolic blood pressure increased in the participants during the mental arithmetic test. However, regression analysis revealed that systolic blood pressure is not a significant predictor of BHD during mental arithmetic tasks. Regarding diastolic blood pressure and its relation to BHD, it showed a significant and negative correlation with BHD (P < 0.05) during resting conditions.

**Table 3:** Relationship between breath holding duration (BHD), which is the dependent variable with Systolic BP, Diastolic BP, & Oxygen saturation (SpO2) among the same subject

Situations		Unstandardized Coefficients		t	P-value

		β	Std. Error		
**At rest**	Systolic BP	0.402	0.143	2.802	0.006**
	Diastolic BP	-0.524	0.204	-2.575	0.012*
	Oxygen saturation (SpO2)	20.045	13.935	1.438	0.154
**During the isometric handgrip test**	Systolic BP	0.269	0.095	2.821	0.006**
	Diastolic BP	-0.044	0.107	-0.413	0.681
	Oxygen saturation (SpO2)	-6.244	9.555	-0.653	0.515
**During the cold pressor test**	Systolic BP	0.250	0.103	2.424	0.018*
	Diastolic BP	-0.220	0.128	-1.711	0.091
	Oxygen saturation (SpO2)	18.372	11.514	1.596	0.115
**During mental arithmetic task**	Systolic BP	0.178	0.125	1.428	0.157
	Diastolic BP	0.018	0.153	0.114	0.909
	Oxygen saturation (SpO2)	8.977	10.964	0.819	0.415

** Significant at %

* Significant at 5%

There were no significant changes in diastolic blood pressure during the isometric handgrip, CPT, and mental arithmetic tests. Additionally, no significant alterations were observed in the SpO2 levels of participants in any of the experimental situations studied. During the isometric handgrip test, we found that systolic blood pressure was the only significant predictor of breath-holding duration (Table 3). An increase of one unit in systolic blood pressure led to an increase of 26.9% in BHD. Our findings showed that systolic blood pressure and diastolic blood pressure were significantly affected by the BHD; one unit increase in systolic blood pressure led to about a 40.2% increase in BHD, one unit increase in diastolic blood pressure led to about a 52.4% decrease in BHD.

## Discussion

This research examined the determinants affecting both BHD and the breaking point. The isometric handgrip test revealed a noteworthy reduction in BHD compared to the resting condition. Additionally, this study found a significant increase in BHD while performing the cold pressor and mental arithmetic tests. Moreover, there were no significant differences in SpO2 levels while performing BHD in different situations. Lastly, this study found a significant difference in BHD at rest when comparing the blood pressures between subjects.

According to researchers, the physiological mechanism of breath-holding is complex, involving chemoreceptors, mental factors, various respiratory reflexes, and lung stretchability (5). It has been reported that voluntary breath-holding stimulates both peripheral and central chemoreceptors (16). It is a well-established fact that voluntary breath-holding increases arterial carbon dioxide and decreases oxygen levels. It further leads to chemoreceptor stimulation and initiation of breathing, where the breaking point is achieved (1).

In this study, the BHD of subjects was prolonged during CPT. One possible hypothesis for our finding is that when the subject's palm is immersed in an ice-cold water bath during CPT, it might lead to peripheral vasoconstriction. One of the known mechanisms that increases cerebral blood flow (CBF) is peripheral vasoconstriction (17).

Researchers have shown alpha-adrenoreceptors' involvement in inducing cutaneous vasoconstriction during CPT (18). Studies have also reported that voluntary breath-holding results in cerebral vasodilation and a subsequent increase in CBF(19). The increased CBF eventually washes out the carbon dioxide in the brain's central chemoreceptors, reducing the drive for breathing (20). We assume that in this study, CPT might have reduced the urge to breathe and increased BHD, possibly through these mechanisms. This study revealed a decrease in BHD during the isometric hand grip test, possibly due to increased skeletal muscle activity in the forelimb causing peripheral vasodilation (21) and reduced blood flow to the brain. Our hypothesis is also strengthened by a research report which showed a decrease in CBF after a one-minute sustained isometric handgrip exercise (22); their study was based on transcranial Doppler ultrasound measurement of the middle cerebral artery during isometric hand grip testing. We postulate that a diminished CBF might have triggered the central chemoreceptors, increased the respiratory drive, and consequently reduced BHD.

This study found that BHD increased while performing mental arithmetic tasks. Researchers have reported the link between mental status and its influence on BHD (6). The present study is in accordance with earlier reports that suggest an elevation in BHD during mental arithmetic tasks or engaging in mental distracting activities, such as squeezing a ball (5). Our investigation similarly observed a significant increase in BHD during the mental arithmetic test, supporting these prior findings. Regarding SpO2 levels, we could not find any significance in their relationship with BHD. This observation aligns with a previous report where SpO2 levels did not change during voluntary breath-holding in health subjects (23). Voluntary breath-holding during the isometric hand grip task in our study revealed an increase in systolic blood pressure and diastolic pressure. This finding aligns with a previous report where systolic and diastolic blood pressure increased during voluntary breath-holding (24). This increase in systolic and diastolic blood pressure observed could be attributed to the stimulation of central and peripheral chemoreceptors by increased arterial carbon dioxide during voluntary breath-holding (16). Baroreceptors are mechanoreceptors located within the carotid sinuses and the aortic arch responsible for sensing changes in blood pressure. It has been reported that breath-holding induces baroreceptor stimulation due to the rise in the mean arterial blood pressure, which causes reflex bradycardia (25). This study found that subjects with high resting systolic blood pressure and low resting diastolic pressure could hold their breath longer. Although we could not give a conclusive explanation for this observation, we believe these changes are due to multifactorial elements affecting BDH. We posit that certain changes may indicate gender distinctions, given that this study encompassed both male and female participants. Cardiovascular reactivity and alterations in autonomic function due to hormones like estrogen and progesterone during the menstrual cycle are well documented (26). Researchers have shown increased parasympathetic activity during the follicular phase and enhanced sympathetic activity during the luteal phase of the menstrual cycle. BHD was also found to be higher in the mid-luteal phase of the menstrual cycle (26). The menstrual phase-related variations in autonomic functions could be one of the reasons for the observed link between BHD systolic and diastolic blood pressures.

Psychological factors have been shown to impact BHD (5,6). Consequently, the mental state of the subjects may be a factor that could affect the results of the BHD. However, our study was unable to determine the mental status of the subjects, such as their anxiety levels or stress, during the performance of the tests.

In conclusion, our investigation observed a shorter BHD during the isometric handgrip test, whereas it was longer during both the CPT and mental arithmetic tasks. Additionally, breath-holding did not affect SpO2 during any experiment stage. Although there are variations in methodologies across different studies, this is the first report correlating BHD with the isometric handgrip test, CPT, and mental arithmetic test. We believe that the results of our research have shed some light on some of the factors that could affect BHD. Our finding adds new knowledge and dimensions to the complex respiratory physiology of voluntary breath-holding and various factors affecting it.

## References

[1] Parkes MJ (2006). Breath-holding and its breakpoint. Exp Physiol.

[2] Pernett F, Bergenhed P, Holmström P, Mulder E, Schagatay E (2023). Effects of hyperventilation on oxygenation, apnea breaking points, diving response, and spleen contraction during serial static apneas. Eur J Appl Physiol.

[3] Bagavad GM, Roopa S, Subhashini AS, Syed Suithan K (2014). Effect of physical training on breath holding time in Indian subjects. Indian J Physiol Pharmacol.

[4] Broaddus V Courtney (2016). Murray & Nadel's Textbook of Respiratory Medicine.

[5] Skow PJ, Day TA, Fuller JE, Bruce CD, Steinback CD (2015). The ins and outs of breath holding: simple demonstrations of complex respiratory physiology. Adv Physiol Educ.

[6] Vigran HJ, Kapral AG, Tytell ED, Kao MH (2019). Manipulating the perception of time affects voluntary breath-holding duration. Physiol Rep.

[7] Melnychuk MC, Dockree PM, O'Connell RG, Murphy PR, Balsters JH, Robertson IH (2018). Coupling of respiration and attention via the locus coeruleus: Effects of meditation and pranayama. Psychophysiology.

[8] Watso JC, Cuba JN, Boutwell SL (2023). Acute nasal breathing lowers diastolic blood pressure and increases parasympathetic contributions to heart rate variability in young adults. Am J Physiol Regul Integr Comp Physiol.

[9] Han Y, Du J, Wang J (2022). Cold Pressor Test in Primary Hypertension: A Cross-Sectional Study. Front Cardiovasc Med.

[10] Watts AC, Naylor LA, Jensen BT, Holmstrup ME (2023). The Impact of the Cold Pressor Test on Inter-arm Differences in Blood Pressure. Int J Exerc Sci.

[11] Zhu R, Li W, Xia L (2020). Hand grip strength is associated with cardiopulmonary function in Chinese adults: Results from a cross-sectional study. J Exerc Sci Fit.

[12] Charan J, Biswas T (2013). How to calculate sample size for different study designs in medical research?. Indian J Psychol Med.

[13] Benton MJ, Spicher JM, Silva-Smith AL (2022). Validity and reliability of handgrip dynamometry in older adults: A comparison of two widely used dynamometers. PLoS One.

[14] Silverthorn DU, Michael J (2013). Cold stress and the cold pressor test. Adv Physiol Educ.

[15] Kume D, Nishiwaki M, Hotta N, Endoh H (2022). Impact of acute mental stress on ankle blood pressure in young healthy men: a pilot study. BMC Res Notes.

[16] Bruce CD, Vanden Berg ER, Pfoh JR, Steinback CD, Day TA (2021). Prior oxygenation, but not chemoreflex responsiveness, determines breath-hold duration during voluntary apnea. Physiol Rep.

[17] Kiyatkin EA (2021). Functional role of peripheral vasoconstriction: not only thermoregulation but much more. J Integr Neurosci.

[18] Frank SM, Raja SN (1994). Reflex cutaneous vasoconstriction during cold pressor test is mediated through alpha-adrenoceptors. Clin Auton Res.

[19] Kastrup A, Li TQ, Glover GH, Moseley ME (1999). Cerebral blood flow-related signal changes during breath-holding. AJNR Am J Neuroradiol.

[20] Bain AR, Ainslie PN, Hoiland RL (2016). Role of cerebral blood flow in extreme breath holding. Transi Neurosci.

[21] A Correia M, Oliveira PL, Farah BQ (2020). Effects of Isometric Handgrip Training in Patients With Peripheral Artery Disease: A Randomized Controlled Trial. J Am Heart Assoc.

[22] Hadzic V, Ruzic L, Matkovic B (2021). Decrease In Cerebral Blood Flow During Maximal Handgrip Isometric Contraction–A Brief Report. Kinesiology.

[23] Parkes MJ, Green S, Stevens AM, Clutton-Brock TH (2014). Assessing and ensuring patient safety during breath-holding for radiotherapy. Br J Radiol.

[24] Watanabe H, Washio T, Saito S, Ogoh S (2022). Effect of breath-hold on the responses of arterial blood pressure and cerebral blood velocity to isometric exercise. Eur J Appl Physiol.

[25] Reyes del Paso GA, Muñoz Ladrón de Guevara C, Montoro CI (2015). Breath-Holding During Exhalation as a Simple Manipulation to Reduce Pain Perception. Pain Med.

[26] Cherouveim ED, Botonis PG, Tsakiris T, Koskolou MD, Geladas ND (2020). The effect of menstrual cycle on maximal breath-hold time. Respir Physiol Neurobiol.

